# Microstructure Design of High-Entropy Alloys Through a Multistage Mechanical Alloying Strategy for Temperature-Stable Megahertz Electromagnetic Absorption

**DOI:** 10.1007/s40820-022-00886-6

**Published:** 2022-07-09

**Authors:** Xiaoji Liu, Yuping Duan, Yuan Guo, Huifang Pang, Zerui Li, Xingyang Sun, Tongmin Wang

**Affiliations:** 1 Key Laboratory of Solidification Control and Digital Preparation Technology, School of Materials Science and Engineering, Dalian University of Technology, Dalian, Liaoning 116085 People’s Republic of China; 2 School of Physics, Dalian University of Technology, Dalian, Liaoning 116024 People’s Republic of China

**Keywords:** Electromagnetic wave absorption, Multistage mechanical alloying, High-entropy alloys, Temperature-stable, Corrosion resistance

## Abstract

Developing megahertz (MHz) electromagnetic wave (EMW) absorption materials with broadband absorption, multi-temperature adaptability, and facile preparation method remains a challenge. Herein, nanocrystalline FeCoNiCr_0.4_Cu_0.2_ high-entropy alloy powders (HEAs) with both large aspect ratios and thin intergranular amorphous layers are constructed by a multistage mechanical alloying strategy, aiming to achieve excellent and temperature-stable permeability and EMW absorption. A single-phase face-centered cubic structure with good ductility and high crystallinity is obtained as wet milling precursors, via precisely controlling dry milling time. Then, HEAs are flattened to improve aspect ratios by synergistically regulating wet milling time. FeCoNiCr_0.4_Cu_0.2_ HEAs with dry milling 20 h and wet milling 5 h (D20) exhibit higher and more stable permeability because of larger aspect ratios and thinner intergranular amorphous layers. The maximum reflection loss (*RL*) of D20/SiO_2_ composites is greater than − 7 dB with 5 mm thickness, and EMW absorption bandwidth (*RL* < − 7 dB) can maintain between 523 and 600 MHz from − 50 to 150 °C. Furthermore, relying on the “cocktail effect” of HEAs, D20 sample also exhibits excellent corrosion resistance and high Curie temperature. This work provides a facile and tunable strategy to design MHz electromagnetic absorbers with temperature stability, broadband, and resistance to harsh environments.

**Supplementary Information:**

The online version contains supplementary material available at 10.1007/s40820-022-00886-6.

## Highlights

- Nanocrystalline high-entropy alloy powders (HEAs) with the unprecedented combination of superior manufacturability, large aspect ratios, and thin intergranular amorphous layers are constructed via a multistage mechanical alloying strategy.
- FeCoNiCr_0.4_Cu_0.2_ HEAs for 20 h of dry milling and 5 h of wet milling (D20) exhibit excellent corrosion resistance, high Curie temperature, and temperature-stable broadband megahertz electromagnetic wave absorption.

**Supplementary Information:**

The online version contains supplementary material available at 10.1007/s40820-022-00886-6.

## Graphical abstract

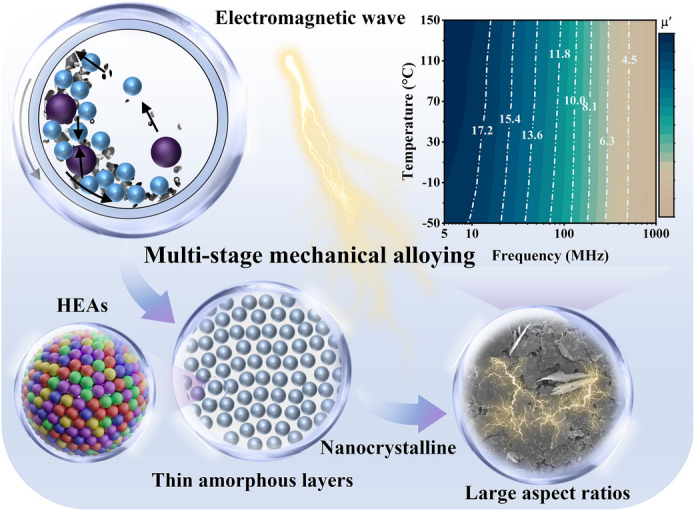

## Introduction

Electromagnetic wave (EMW) absorption materials are ubiquitously used in military, aerospace, communication, and electronic industries to achieve military stealth, shielding electromagnetic interference, and prevent EMW pollution [1–11]. Today, most electronic devices still operate in the megahertz (MHz) frequency band. Facing longer wavelengths than gigahertz EMW, absorbers demand larger and more matched complex permittivity and complex permeability to achieve broadband EMW absorption in the MHz band [12–16]. In addition, the harsh service environments bring challenges to the multi-temperature adaptability (at − 50–150 °C) and corrosion resistance of EMW absorbers [13, 17–22]. Excellent permeability and its temperature stability are of a paramount significance for achieving broadband and temperature-stable EMW absorption in the MHz frequency range [13, 23]. Iron–cobalt–nickel-based high-entropy alloy powders (HEAs) prepared by mechanical alloying method (or high-energy ball milling method) are suitable candidates for EMW absorption materials serving in the MHz band and a wide temperature spectrum, which exhibit several advantages. (1) Excellent magnetic properties with high saturation magnetization, low coercivity, high complex permeability, and high Curie temperature [13, 24]. (2) High resistivity due to severe lattice distortion effects [24, 25]. The high resistivity can help to reduce the complex permittivity to improve impedance matching and EMW absorption performance. (3) Superior manufacturability of the HEAs with large aspect ratios and nanocrystalline structure at the same time [13, 26]. The nanocrystalline structure and large aspect ratios facilitate the averaging of magnetocrystalline anisotropy and the breaking of the Snoek’s limit [23, 27, 28], respectively. This facilitates the improvement of the permeability and its temperature stability over a wide temperature range. Moreover, mechanical alloying method has the advantages of room-temperature processing, homogenous element distribution, energy saving, and high efficiency [29–31], as compared with the complicated process commonly used for HEAs, such as carbothermal shock synthesis [32], ultrafast-cooling arc-discharged approach [33, 34], atomization [31], and melting-strip casting–milling [26].

In the past, our group has successfully prepared flake-shaped FeCoNiCr_0.4_Cu_0.2_ HEAs with nanocrystalline and thin amorphous layers nanostructure by mechanical alloying method to break the Snoek’s limit and enhance intergranular magnetic coupling. FeCoNiCr_0.4_Cu_0.2_ HEAs with this special structure exhibit excellent and temperature-stable permeability and EMW absorption performance in the MHz frequency range at − 50–150 °C [13, 23]. To obtain flake-shaped HEAs to enhance planar anisotropy and break the Snoek’s limit, anhydrous ethanol is usually added as a process control agent [25, 35]. During the mechanical alloying process, anhydrous ethanol adsorbs on the surface of metal particles and reduces the surface energy to inhibit powder agglomeration and obtain flake-shaped HEAs [25]. It also enables the HEAs to avoid sticking to the ball and tank surface contributing to high yield. Nevertheless, the addition of anhydrous ethanol leads to a continuous refinement of the particle size and a steady-state size distribution at a small particle size for HEAs accompanying repeated cold welding and fracturing. This eventually limits the regulation of the aspect ratio [26, 31]. In addition, anhydrous ethanol also decomposes during the high-energy ball milling process causing the introduction of carbon and oxygen impurities [25, 29, 30], which is not conducive to reducing the coercivity and improve permeability.

Compared with the wet milling method (anhydrous ethanol as process control agents), the HEAs prepared by the dry milling method (without anhydrous ethanol) have larger particle sizes, better elemental homogeneity, and greater permeability [26]. Simultaneously, dry milling method can also reduce the introduction of impurities and greatly improve the efficiency of alloying. However, according to the classic mechanisms of mechanical alloying, HEAs can absorb more energy for plastic deformation and lattice distortion during the dry milling process. This eventually leads to severe amorphization and thick intergranular amorphous layers [25]. This is not conducive to maintaining the magnetic coupling between nanograins and stable permeability at elevated temperature. Therefore, the trade-off between large aspect ratios, nanocrystalline structure with thin intergranular amorphous layers, and manufacturability has been a major challenge. The ideal solution to solve this dilemma is to rationally design the mechanical alloying process parameters to precisely regulate the microstructure and micromorphology of HEAs. Among the mechanical alloying parameters, the milling time crucially determines the phases, grain sizes, and particle sizes of HEAs [29].

In this work, we innovatively design a multistage mechanical alloying (MMA, dry milling first, then wet milling) strategy to construct nanocrystalline HEAs with large aspect ratios and thin intergranular amorphous layers, aiming to achieve excellent and temperature-stable permeability and broadband MHz electromagnetic absorption. Our design concept aims at modulating the phase transition to obtain a single-phase face-centered cubic (FCC) structure with superior ductility and high crystallinity as wet milling precursors, via precisely controlling the dry milling time. Then, HEAs precursors are flattened to improve aspect ratios by synergistically regulating wet milling time. Meanwhile, we reveal the phase transition mechanism during multistage mechanical alloying process and provide a new strategy to design the microstructure and micromorphology of HEAs for EMW absorption and magnetic applications in electrical industries.

## Experiments

### Preparation of HEAs

**Fig. 1:**
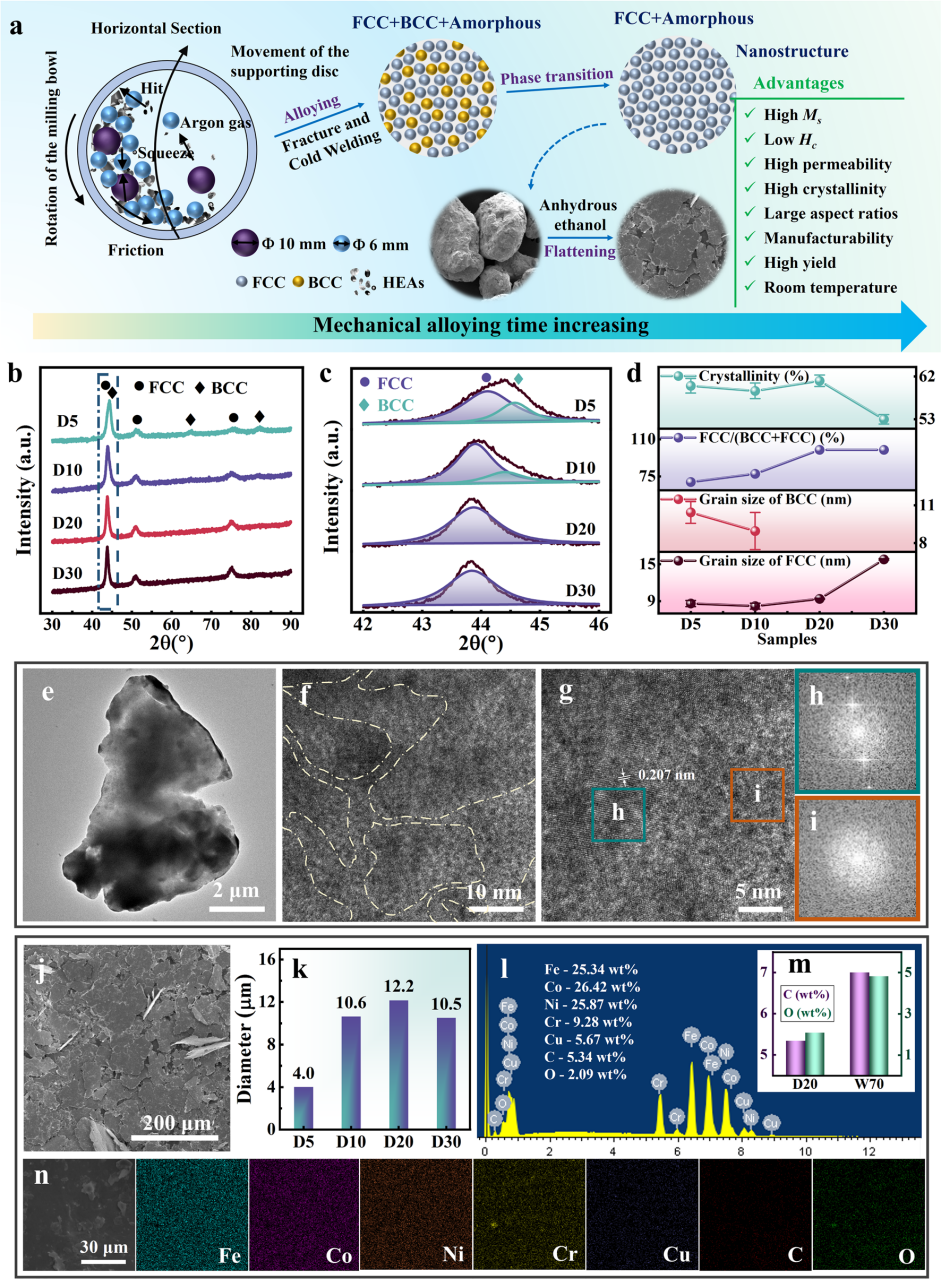
**a** Multistage mechanical alloying strategy and microstructure design concept. **b** XRD and **c** partial enlarged image, **d** grain size, FCC phase ratio, and crystallinity chart of D5, D10, D20, and D30 samples. **e** TEM image of D20 sample. **f, g** HRTEM image of D20 sample and **h, i** the corresponding FFT images of nanocrystalline and amorphous regions, respectively. **j** SEM micrograph of D20 sample. **k** Length-weighted average diameters of D5, D10, D20, and D30 samples. **l** EDS energy spectrum of D20 sample and **m** mass ratios of carbon and oxygen elements in D20 and W70 samples. **n** Elements mapping of D20 sample

The spherical metal powders of Fe, Co, Ni, Cr, and Cu with particle sizes less than 50 μm and purity greater than 99% are used as raw materials for the preparation of FeCoNiCr_0.4_Cu_0.2_ (atomic ratio) HEAs (Table S1). All FeCoNiCr_0.4_Cu_0.2_ HEAs are prepared in a QM-QX2 ball mill by mechanical alloying. The mass ratio of ball to metal powders is 20:1, and the mass ratio of large ball (10 mm) to small ball (6 mm) is 1:2. Four minutes pause are set every half hour during operation to prevent overheating. Multistage mechanical alloying (MMA) strategy adopts the process of first dry milling (without anhydrous ethanol, argon atmosphere) and then wet milling (add 40 mL anhydrous ethanol as a process control agent) to prepare FeCoNiCr_0.4_Cu_0.2_ HEAs. The prepared samples are named as D5 (dry milling 5 h, wet milling 5 h), D10 (dry milling 10 h, wet milling 5 h), D20 (or W5, dry milling 20 h, wet milling 5 h), D30 (dry milling 30 h, wet milling 5 h), W3 (dry milling 20 h, wet milling 3 h), W8 (dry milling 20 h, wet milling 8 h), and W10 (dry milling 20 h, wet milling 10 h), respectively. We also prepare comparison samples for 20 h of dry milling only and 70 h of wet milling (W70) only for exploring the evolution mechanism of FeCoNiCr_0.4_Cu_0.2_ HEAs microstructure. The detailed preparation process of MMA strategy and microstructure design concept are shown in Fig. 1a.

### Structure and Morphology

The phase structure of FeCoNiCr_0.4_Cu_0.2_ HEAs is characterized by a X-ray diffraction (XRD, D8 Advance) with Cu-Kα radiation (the step size is 0.02°; the residence time is 0.5 s). We use a field emission transmission electron microscope (TEM, JEM-F200) to characterize the microstructure of FeCoNiCr_0.4_Cu_0.2_ HEAs. We examine the microscopic morphology, element distribution and EDS energy spectrum of FeCoNiCr_0.4_Cu_0.2_ HEAs by a scanning electron microscope (SEM, SUPRA55) with energy-dispersive spectrometer (EDS) detector. The length-weighted average diameters of FeCoNiCr_0.4_Cu_0.2_ HEAs are tested by the laser scattering particle size distribution analyzer (BT-9300ST) for comparing the aspect ratios.

### Electromagnetic Properties

The vibrating sample magnetometer (VSM7404-S) is used to measure the coercivity *H*_*c*_ and saturation magnetization *M*_*s*_ of D5, D10, D20, and D30 samples from 30 to 150 °C under a applied field of 12,000 Oe. The Curie temperature of D20 sample is also measured with the VSM from 50 to 840 °C. We measure the complex permeability and complex permittivity of samples at − 50–150 °C by Keysight E4991B impedance analyzer with Espec SU-262 Bench-top temperature chamber. The pure D5, D10, D20, and D30 samples are prepared into a concentric ring with inner diameter of 3.1 mm and outer diameter of 8 mm to investigate the permeability temperature dependence. Moreover, the D20 sample and SiO_2_ are uniformly mixed according to the mass ratios of 9:1, 8:2, 7:3, and 6:4 and then pressed into concentric rings with inner diameter of 3.1 mm and outer diameter of 8 mm to measure the complex permeability and disk with diameter of 18 mm to measure complex permittivity for calculating reflection loss. Finally, we use D20/SiO_2_ composites with mass ratio of 8:2 to test the complex permeability and complex permittivity at − 50–150 °C to calculate reflection loss at different temperatures.

### Corrosion Resistance Properties

An electrochemical workstation (M204, Metrohm Autolab) is used to test the potentiodynamic polarization curve and electrochemical impedance spectra (EIS) of D20 sample in the 3.5 wt% NaCl solution. The electrochemical tests adopt a three-electrode system with a saturated calomel electrode (SCE) as the reference electrode, a platinum sheet as the counter electrode, and the D20 sample as the working electrode with an exposed area of 1 cm^2^ (pressed under 30 MPa pressure). The open-circuit potential of D20 sample is obtained after being soaked for 1800 s. The potentiodynamic polarization curve is measured from the cathodic potential to the anodic potential with the scanning rate of 0.5 mV s^−1^. The EIS test is carried out with a scan amplitude of 5 mV and frequencies ranging 100 kHz to 0.1 Hz. Data processing for the electrochemical tests is completed by software NOVA2.1 which is equipped with the workstation. Corrosion potential and corrosion current density are obtained from polarization curve.

## Results and Discussions

### Microstructure Design and Characterization

Mechanical alloying is a potential synthesis technique for the production of flake-shaped HEAs with nanocrystalline structure [29, 30]. During the mechanical alloying process, the metal particles undergo repeated fracturing and cold welding with the synergistic effects of centrifugal force, hit force, squeeze force and friction force and are finally welded together. Alloying of metal particles at the atomic level is achieved through reduced diffusion distances, increased defect density, and heat released during ball milling. At the same time, under the action of various stresses, the metal particles produce severe plastic deformation and high density of dislocations. The annihilation and rearrangement of dislocations form subgrain boundaries. Nanocrystallines eventually form through grain boundary slip or grain rotation [29]. Therefore, the difference from other alloying techniques is that mechanical alloying has the advantages of achieving alloying at room temperature, excellent element solid solution ability, and element uniformity. The phase formation mechanism of mechanical alloying is also different from other alloying techniques. Elements with the same crystal structure are more likely to be solid-dissolved and maintain the original phase structure. For instance, Cr element is easy to dissolve into Fe element and form a body-centered cubic (BCC) structure, Cu element is easy to solid-dissolve into Ni element and form a FCC structure, and Co element is present in both FCC and BCC structures of FeCoNiCr_0.4_Cu_0.2_ HEAs [23]. According to previous studies, the FCC phase has more slip directions than the BCC phase, which enables it to be better ductility and fracture toughness to maintain grain integrity and large particle sizes during mechanical alloying [36–38]. Therefore, in order to obtain FeCoNiCr_0.4_Cu_0.2_ HEAs with high crystallinity and large aspect ratios to enhance the permeability and its temperature stability, we design a MMA strategy, aiming to achieve alloying, phase transition, and large particle sizes by dry milling and flattening of alloy particles by wet milling, as shown in Fig. 1a.

The XRD image shows that the five metal elements are fully alloyed and form simple solid solution structure with FCC phase (corresponding to (111), (200), and (220)) and BCC phase (corresponding to (110), (200), and (211)) after 5 h of dry milling and 5 h of wet milling, as shown in Fig. 1b. Figure 1c is a partial enlarged and split-peak fit picture of XRD from 42° to 46°, it shows that the diffraction peaks corresponding to BCC phase gradually broaden and disappear, and the diffraction peaks of FCC phase remain basically unchanged with the dry milling time increasing. To further explore the phase transformation mechanism of FeCoNiCr_0.4_Cu_0.2_ HEAs during the alloying process, we calculate the grain size by the Williamson–Hall formula (Eq. S1) [39], calculate the phase ratio by the XRD diffraction peak area ratio [35], and perform peak shape fitting with Jade 6 (MDI) to calculate the crystallinity [25]. Figure 1d shows that the grain size of BCC phase decreases from 10.4 to 9 nm until it disappears, and the grain size of FCC phase decreases from 8.6 to 8.2 nm, then increases to 9.4 nm, and finally increases sharply to 15.7 nm with dry milling time increasing. At the same time, the proportion of FCC phase gradually becomes larger with dry milling time increasing. The crystallinity remains basically constant at 5–20 h of dry milling and decreases significantly at 30 h of dry milling. Overall, as the dry milling time increasing from 5 to 20 h, the grains of the BCC phase gradually decrease until disappear under severe plastic deformation, and the amorphous phase transforms into the FCC phase. The transformation to FCC phase is completed after 20 h of dry milling, and the crystallinity of FeCoNiCr_0.4_Cu_0.2_ HEAs decreases with dry milling time further increasing. Thus, the D20 sample has simultaneously a single-phase FCC structure and a high crystallinity. In addition, Fig. S1 shows that the single-phase FCC structure has been formed after only 20 h of dry milling, which indicates that the phase transition mainly occurs in the dry milling stage. The microstructure of D20 sample is further characterized by HRTEM (Fig. 1f-g). We find that the D20 sample is unique nanocrystalline structure (Fig. 1h) separated by amorphous layers (Fig. 1i). The lattice fringe with a lattice spacing of 0.207 nm is consistent with the (111) facet of FCC phase. The average intergranular amorphous layer thickness Λ of D5, D10, D20, and D30 samples is calculated by the following formula [40–42]:

1 $${\wedge}= \overline{D}\left[ {\left( {1/V_{cr} } \right)^{1/3} - 1} \right]$$

where Λ is the average intergranular amorphous layer thickness, $\overline{D }$ is the average grain size, and *V*_*cr*_ is the crystalline volume fraction. Table 1 shows that the average intergranular amorphous layer thickness of the D20 sample is less than 2 nm, which is attributed to the small grain size and high crystallinity. The thin intergranular amorphous layers facilitate enhancing magnetic coupling between nanograins and temperature stability of permeability at different temperatures [13, 27]. The TEM (Fig. 1e) and SEM (Fig. 1j) images show that the D20 sample has a flake-shaped structure and thin thickness. The D20 sample has a larger particle size compared to other samples especially the W70 sample (Fig. S2). Combined with Fig. S3, it is easy to find that the HEAs are large spherical particles after only 20 h of dry milling. This indicates that the alloy particles are flattened by wet milling. Here, we use the length-weighted average diameters to represent the aspect ratios. Figure 1k shows that the D20 sample has larger aspect ratios compared to other samples with different dry milling times, which is attributed to the good ductility and fracture toughness of the single-phase FCC structure and matching wet milling time. Compared to other samples with different wet milling times, the D20 sample also has the larger aspect ratios, as shown in Fig. S4. This shows that the alloy particles are not sufficiently flattened when the wet milling time is short, and the alloy particles are seriously crushed when the wet milling time is long. In addition, Fig. 1m shows that the HEAs prepared by MMA have less carbon and oxygen elements contamination, which is beneficial to reduce coercivity and improve permeability. The elements mapping of Fe, Co, Ni, Cr, and Cu elements shows that the HEAs prepared by MMA have better element uniformity and stronger solid solution ability, as shown in Figs. 1n and S6.

**Table 1:** The grain size of FCC phase *D*_*FCC*_, grain size of BCC phase *D*_*BCC*_, FCC phase ratio, BCC phase ratio, average grain size *D*_*aver*_, *crystallinity* and average intergranular amorphous layer thickness Λ of D5, D10, D20, and D30 samples

Samples	D_FCC_ (nm)	D_BCC_ (nm)	FCC (%)	BCC (%)	*D*_aver_ (nm)	Crystallinity (%)	Λ (nm)
D5	8.6 ± 0.5	10.4 ± 0.9	69.8	30.2	9.2 ± 0.6	60.0 ± 1.5	1.7 ± 0.2
D10	8.2 ± 0.5	9.0 ± 1.5	77.4	22.6	8.4 ± 0.7	59.0 ± 1.6	1.6 ± 0.2
D20	9.4 ± 0.1	–	100	0	9.4 ± 0.1	61.1 ± 1.2	1.7 ± 0.1
D30	15.8 ± 0.1	–	100	0	15.8 ± 0.1	53.0 ± 1.0	3.7 ± 0.1

### Magnetic Performance

**Fig. 2:**
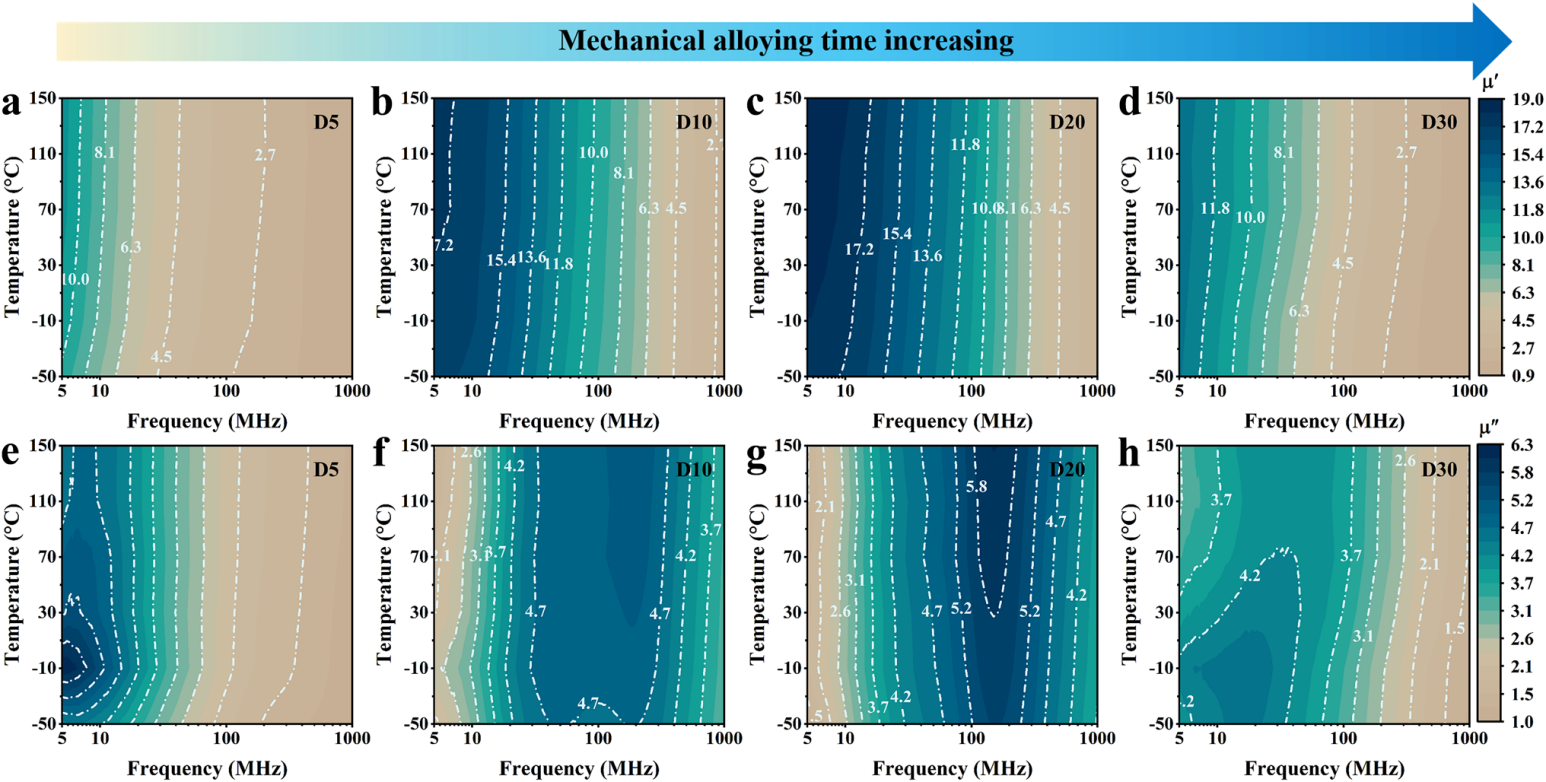
**(a–d**) Real permeability *μ*′ and (**e–h**) imaginary permeability *μ*″ of D5, D10, D20, and D30 samples from − 50 to 150 °C in 5 −100 MHz frequency range

The complex permeability of D5, D10, D20, and D30 samples is measured by impedance analyzer (E4991B) in the MHz band at − 50–150 °C. Figure 2a-d and e–h is the real *μ*′ and imaginary *μ*″ permeability of samples, respectively. Figure 2a-d shows that the real permeability of D5, D10, D20, and D30 samples decreases with the frequency increasing. This is because the natural resonance of D5, D10, D20, and D30 samples occurs in the MHz band resulting in the drop of real permeability and a natural resonance peak in the imaginary permeability. Meanwhile, as increasing frequency, the imaginary permeability of D5, D10, D20, and D30 samples increases and then decreases (Fig. 2e-h). Figure 2a-h shows that the real permeability and imaginary permeability of D5, D10, D20, and D30 samples increase slightly and have excellent temperature stability with the temperature increasing. In particular, D20 sample has more stable complex permeability with the temperature increasing than that of others.

**Fig. 3:**
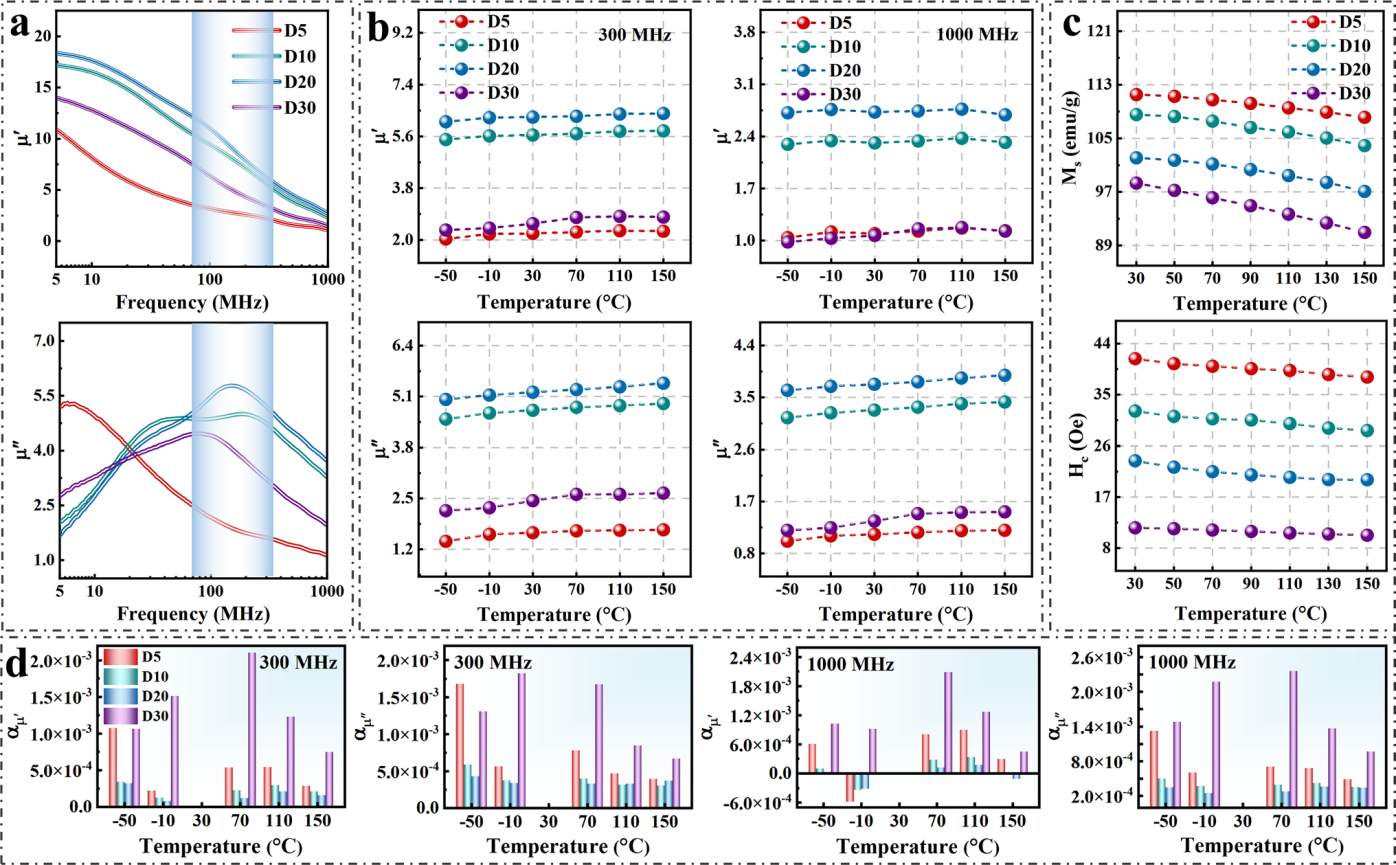
**a** Real permeability *μ*′ and imaginary permeability *μ*″ of D5, D10, D20, and D30 samples in 5−1000 MHz frequency range at room temperature. **b** Real permeability *μ*′ and imaginary permeability *μ*″ of D5, D10, D20, and D30 samples from − 50 to 150 °C at 300 and 1000 MHz, respectively. **c** Saturation magnetization *M*_*s*_ and coercivity *H*_*c*_ of D5, D10, D20, and D30 samples from 30 to 150 °C. **d** Complex permeability temperature coefficient of D5, D10, D20, and D30 samples from − 50 to 150 °C at 300 and 1000 MHz

We compare the complex permeability of D5, D10, D20, and D30 samples in the MHz band at room temperature. As shown in Fig. 3a, the D20 sample has greater real permeability at 5 MHz, which is due to the fact that the permeability is proportional to the saturation magnetization and inversely proportional to the coercivity [27]. Compared to the other samples, the D20 sample has a larger aspect ratio to improve the planer anisotropy and reduce the coercivity, while having a higher crystallinity to maintain a high saturation magnetization (Fig. 3c). In addition, the D20 sample has a larger planer anisotropy to break the Snoek’s limit (Eq. S2) [23], resulting in both great permeability and natural resonance frequency, as shown in Fig. 3a. Compared to W3, W8, and W10 samples, the D20 sample (or W5) also has a greater complex permeability and natural resonance frequency due to the greater aspect ratios, as shown in Fig. S7. To further investigate the complex permeability temperature dependence of D5, D10, D20, and D30 samples, we compare the complex permeability of D5, D10, D20, and D30 samples at 300 and 1000 MHz, respectively, as shown in Fig. 3b. The result shows that the complex permeability of D5, D10, D20, and D30 samples increases slightly and is relatively stable with the temperature increasing. Figure 3c shows that the increase in permeability is mainly due to the decrease in coercivity with the temperature increasing. Because D5, D10, D20, and D30 samples have high crystallinity and thin intergranular amorphous layers to enhance the magnetic coupling between nanograins, they have temperature-stable coercivity and complex permeability. According to the Eq. S3, we calculate the permeability temperature coefficient α_μ_ to represent the permeability temperature stability. The closer the permeability temperature coefficient is to 0, the more stable the permeability is. Figure 3d shows that the D20 sample has a smaller permeability temperature coefficient and more stable permeability from − 50 to 150 °C. Due to the poor crystallinity, the saturation magnetization of the D30 sample decreases rapidly with the temperature increasing, which leads to the poor permeability temperature stability. Table S2 shows that the D20 sample has a larger complex permeability and a smaller permeability temperature coefficient compared to the other samples, which is attributed to both its larger aspect ratios and higher crystallinity. Simultaneously, the D20 sample has a high Curie temperature (774 °C) due to the thin intergranular amorphous layers and the synergistic effect between the elements, as shown in Fig. S8.

### EMW Absorption and Corrosion Resistance Performance

**Fig. 4:**
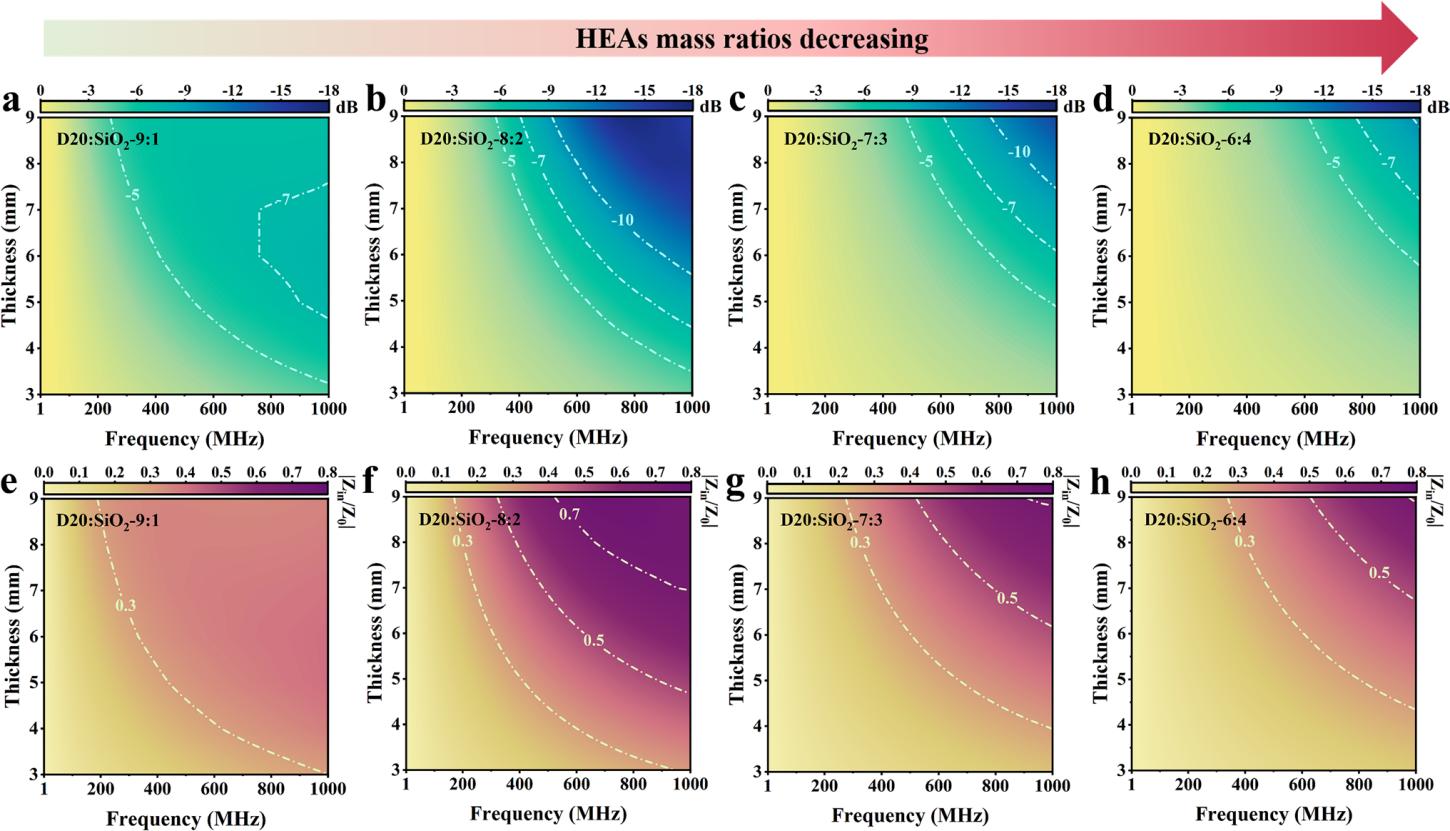
**a–d** Reflection loss and **e–h** impedance matching images of D20/SiO_2_ composites with mass ratios of 9:1, 8:2, 7:3, and 6:4 at room temperature and different thicknesses

D20 sample and SiO_2_ are homogeneously mixed and pressed into D20/SiO_2_ composites under the pressure of 4 MPa to measure the complex permeability and complex permittivity in the MHz frequency range. The reflection loss (*RL*) of D20/SiO_2_ composites at different thicknesses is calculated by the transmission line theory, described as follows [43–46]:

2 $$RL\left( {dB} \right) = 20\log_{10} \left| {\left( {Z_{in} - Z_{0} } \right)/\left( {Z_{in} + Z_{0} } \right)} \right|$$

3 $$\frac{{Z_{in} }}{{Z_{0} }} = \sqrt {\frac{{\mu_{r} }}{{\varepsilon_{r} }}} \tanh \left( {j\frac{2\pi tf}{c}\sqrt {\mu_{r} \varepsilon_{r} } } \right)$$

where Ζ_*in*_ represents the input impedance, Ζ_0_ represents the characteristic impedance of the transmission line, *t* means thickness of absorbers, and *c* represents the speed of light in free space. We adjust the complex permeability and complex permittivity by varying the mass ratios of D20 sample and SiO_2_, aiming at better impedance matching and EMW absorption performance. Figure S9 shows that the complex permeability and complex permittivity of the D20/SiO_2_ composites decrease with the SiO_2_ mass ratio increasing. In particular, the complex permittivity of the D20/SiO_2_ composites with mass ratio of 8:2 is much smaller than the D20/SiO_2_ composites with mass ratio of 9:1, which is beneficial to improve the impedance matching and enhance the EMW absorption performance. Figure 4a-d shows that the D20/SiO_2_ composites with mass ratio of 9:1 have a wider EMW absorption bandwidth (*RL* < − 5 dB, absorption value more than 68.4%). According to the Planck–Rozanov limit (Eq. S4) [47, 48], this is because the D20/SiO_2_ composites with mass ratio of 9:1 have a larger permeability relative to the other mass ratios composites. The D20/SiO_2_ composites with mass ratio of 8:2 have both a wider EMW absorption bandwidth (*RL* < − 7 dB, absorption value more than 80%) and a larger reflection loss, as shown in Fig. 4b. This is because the D20/SiO_2_ composites with mass ratio of 8:2 have large complex permeability and matching complex permittivity to provide better impedance matching performance. The closer the |*Z*_in_*/Z*_0_| is to 1, the better the impedance matching performance, which enables more incident EMW to enter the absorbers without being reflected. Figure 4e–h shows that the impedance matching becomes better and then worse as the mass ratios of HEAs in the D20/SiO_2_ composites decreasing, and the D20/SiO_2_ composites with mass ratio of 8:2 have a better impedance matching.

**Fig. 5:**
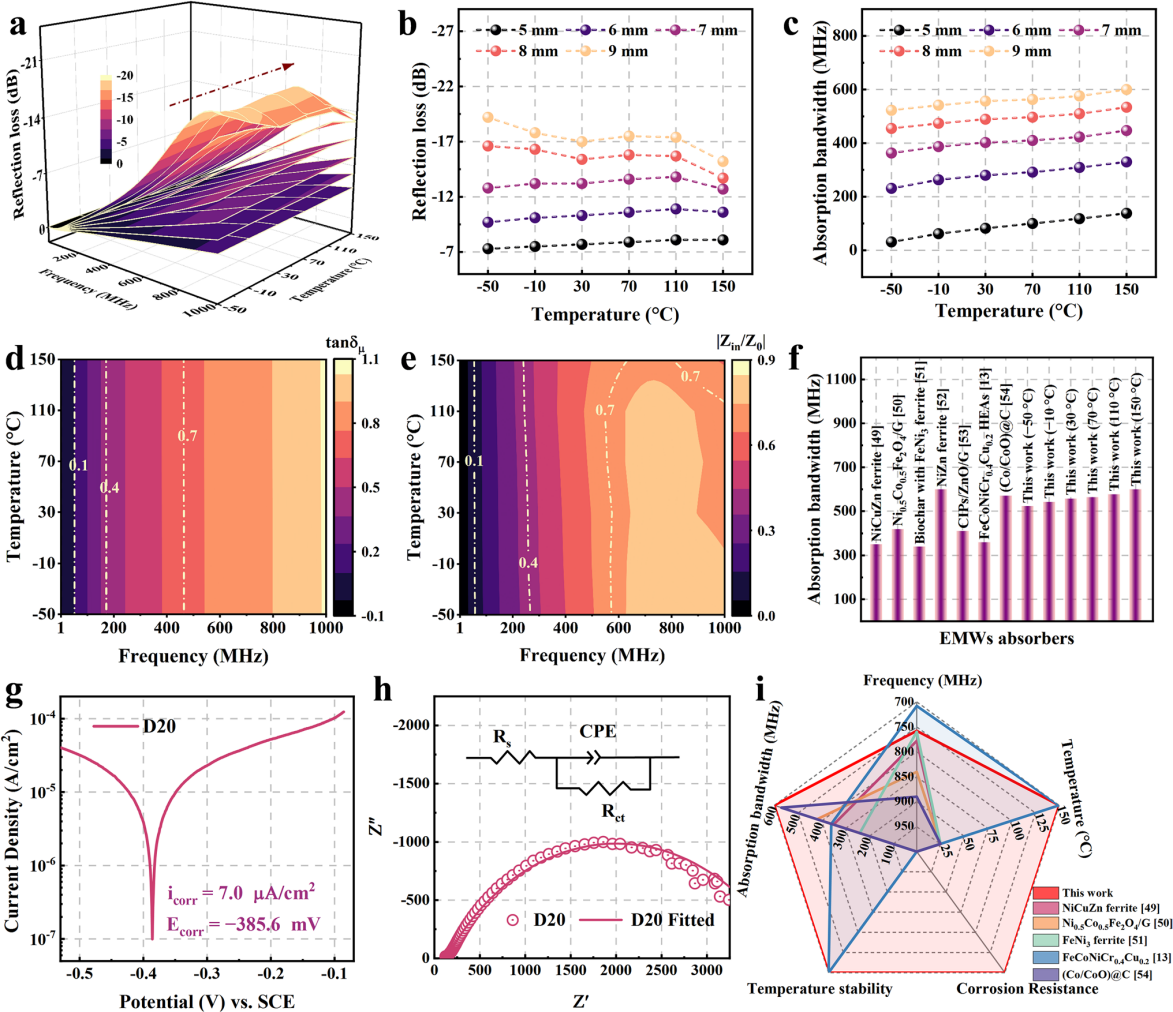
**a** Three-dimensional reflection loss plot of D20/SiO_2_ composites with mass ratio of 8:2 at different thicknesses from − 50 to 150 °C. **b** Maximum reflection loss and **c** absorption bandwidth of D20/SiO_2_ composites with mass ratio of 8:2 at different thicknesses from − 50 to 150 °C, respectively. **d** Magnetic loss tangent of D20/SiO_2_ composites with mass ratio of 8:2 from − 50 to 150 °C. **e** Impedance matching of D20/SiO_2_ composites with mass ratio of 8:2 at 9 mm from − 50 to 150 °C. **f** Comparative chart of EMW absorption bandwidth for MHz EMW absorbers [13, 49–54]. **g** Potentiodynamic polarization curve of D20 sample. **h** Nyquist plots and equivalent circuit of D20 sample. **i** Comparative chart of the comprehensive performance for representative MHz EMW absorbers [13, 49–51, 54]

We measure the complex permeability and complex permittivity of the D20/SiO_2_ composites with 8:2 mass ratio at − 50–150 °C (Fig. S10) to calculate the reflection loss (Fig. S11) and impedance matching (Fig. S12). Figure 5a shows that D20/SiO_2_ composites with 8:2 mass ratio have stable EMW absorption performance at − 50–150 °C, and the maximum reflection loss (*RL*_max_) of the composites shifts to lower frequency with the thickness increasing. To further investigate the temperature dependence of the EMW absorption performance for the D20/SiO_2_ composites, we compare the *RL*_max_ and the absorption bandwidth (*RL* < − 7 dB) with temperature and thickness, respectively. Figure 5b shows that the *RL*_max_ of the D20/SiO_2_ composites becomes larger as the thickness increases. The *RL*_max_ is relatively stable at − 50–110 °C and slightly decreases at 150 °C. This is because the magnetic loss tangent tan*δ*_*μ*_ = *μ*″/*μ*′ (Fig. 5d) and the impedance matching |Ζ_*in*_/Ζ_0_| (Fig. 5e) of D20/SiO_2_ composites are relatively stable at − 50–110 °C. Figure S10 shows that the pemittivity gradually increases with temperature, which leads to poor impedance matching performance (Fig. 5e) and a decrease in EMW absorption performance at 150 °C. When the thickness is 5 mm, the *RL*_max_ is higher than − 7 dB at − 50–150 °C, because of excellent magnetic loss and impedance matching performance. Figure 5c shows that the absorption bandwidth of the D20/SiO_2_ composites becomes wider with the thickness increasing. In addition, the absorption bandwidth slightly increases with the temperature increasing because the permeability of the D20/SiO_2_ composites increases with the temperature increasing, and the absorption bandwidth is proportional to the permeability (Eq. S4). Figure 5f shows the absorption bandwidth of the D20/SiO_2_ composites with 8:2 mass ratio and other MHz EMW absorbers. The results show that the D20/SiO_2_ composites with 8:2 mass ratio have a wider and temperature-stable EMW absorption bandwidth in the MHz frequency range due to the high and temperature-stable permeability [13, 49–54].

Figure 5g displays the potentiodynamic polarization curve of D20 sample. The corrosion current density (icorr) of D20 sample is 7.0 μA cm^−2^. Table S4 shows that the corrosion current density of D20 sample is smaller compared with other HEA [55, 56]. This shows that the D20 sample has excellent corrosion resistance due to the synergistic effect between elements of FeCoNiCr_0.4_Cu_0.2_ HEAs. Figure 5h shows the Nyquist plots and the equivalent circuit of D20 sample in the 3.5 wt% NaCl solution. Generally, the diameter of the semicircle reflects the value of charge transfer resistance (*R*_ct_) [17], the fitted curve is displayed in Fig. 5h, and the fitted values of the equivalent circuit parameters are given in Table S5. The diameter of the semicircle of Nyquist plots and the fitted Rct values (3,739 Ω) of D20 sample are much larger than FeCoNiCu HEAs (2,856 Ω) [57], suggesting that the addition of Cr increases the charge transfer resistance of the passivated film to prevent attack of anions in the NaCl solution. Overall, FeCoNiCr_0.4_Cu_0.2_ HEAs not only have temperature-stable broadband MHz electromagnetic absorption, but also have excellent corrosion resistance (Fig. 5i) [13, 49–51, 54]. This indicates that these HEAs constructed by MMA strategy have the potential to be used in harsh environments.

## Conclusions

In summary, we have successfully developed the nanocrystalline FeCoNiCr_0.4_Cu_0.2_ high-entropy alloy powders (HEAs) with the unprecedented combination of superior manufacturability, homogenous element distribution, large aspect ratios, and thin intergranular amorphous layers by a multistage mechanical alloying (MMA) strategy. Research results show that the phase transition occurs mainly in the dry milling stage during MMA. As the dry milling time increases, the BCC phase gradually transforms into amorphous phase and the amorphous phase transforms into FCC phase. FeCoNiCr_0.4_Cu_0.2_ HEAs with both a single-phase FCC structure and high crystallinity can be obtained by precisely regulating the dry milling time. The flattening process of alloy particles occurs mainly in the wet milling stage. The alloy particles are not sufficiently flattened when the wet milling time is short, and seriously crushed when the wet milling time is long. FeCoNiCr_0.4_Cu_0.2_ HEAs for 20 h of dry milling and 5 h of wet milling (D20 sample) exhibit greater complex permeability and smaller permeability temperature coefficient than that of others because of larger aspect ratios and thinner intergranular amorphous layers. The maximum reflection loss (*RL*) of D20/SiO_2_ composites is greater than − 7 dB (absorption value more than 80%) with 5 mm thickness and EMW absorption bandwidth (*RL* < − 7 dB) can maintain between 523 and 600 MHz from − 50 to 150 °C. Relying on thin intergranular amorphous layers and synergistic effect between elements, the Curie temperature of D20 sample can reach 774 °C. Simultaneously, FeCoNiCr_0.4_Cu_0.2_ HEAs also have excellent corrosion resistance (icorr = 7.0 μA cm^−2^ and *R*_ct_ = 3739 Ω). This indicates that HEAs constructed by MMA strategy have the potential to be used in harsh environments.

## Supplementary Information

Below is the link to the electronic supplementary material.

Supplementary file1 (PDF 1451 kb)
