# Supplementary material for: Microstructure Design of High-Entropy Alloys Through a Multistage Mechanical Alloying Strategy for Temperature-Stable Megahertz Electromagnetic Absorption
Source: Nanomicro Lett. 2022 Jul 9;14:142. doi: 10.1007/s40820-022-00886-6 (PMC9271152; doi:10.1007/s40820-022-00886-6)
Supplement: Supplementary file 1 — Supplementary file1 (PDF 1451 kb) [file 40820_2022_886_MOESM1_ESM.pdf]

Supporting Information for

Microstructure Design of High-Entropy-Alloys Through a Multi-Stage Mechanical Alloying Strategy for Temperature-Stable Megahertz Electromagnetic Absorption

Xiaoji Liu[1], Yuping Duan[1,*], Yuan Guo[2], Huifang Pang[1], Zerui Li[1], Xingyang Sun[1], Tongmin Wang[1,*]

[1]Key Laboratory of Solidification Control and Digital Preparation Technology, School of Materials Science and Engineering, Dalian University of Technology, Dalian, Liaoning 116085, P. R. China

[2]School of Physics, Dalian University of Technology, Dalian, Liaoning 116024, P. R. China

*Corresponding authors. E-mail: duanyp@dlut.edu.cn (Yuping Duan); tmwang@dlut.edu.cn (Tongmin Wang)

S1 Williamson-Hall Formula [S1]

$$\beta \cos\theta = K\lambda/D + \varepsilon(4\sin\theta) \quad \text{(S1)}$$

where β is the full width at half-maximum, θ is the main diffraction peak angle, λ is the X-ray wavelength, $K = 0.89$, D is the average grain size, and ε is the internal strain. $\beta\, cos\,\theta$ and $4\, sin\,\theta$ are the y-axis and x-axis, respectively. The average grain size can be obtained from the intercept of the Y axis.

S2 Shape-dependent Snoek's limit [S2]

$$(\mu_i - 1)f_r^2 = \bar{\gamma}^2 4\pi M_s(H_k + 4\pi M_s N_\perp), N_h = \frac{1 - N_\perp}{2} \quad \text{(S2)}$$

where f_r is natural resonance frequency, $\bar{\gamma}$ is the gyromagnetic factor, $N_\perp$ is the vertical demagnetization factor, N_h is the horizontal demagnetization factor, and H_k is magnetocrystalline anisotropy field.

S3 Temperature Coefficient of Permeability αμ

$$\alpha_\mu = \frac{\mu_x - \mu_0}{(T_x - T_0)\mu_0} \quad \text{(S3)}$$

where μ_x is permeability at T_x (x = −50, −10, 30, 70, 110, and 150 °C) and μ_0 is permeability at T_0 (30 °C). The closer permeability temperature coefficient α_μ is to 0, the more stable the permeability with increasing temperature.

S4 Planck-Rozanov Limit [S3, S4]

$$\Delta\lambda < \frac{2\pi^2 \mu_i t}{|ln\, \rho_0|} \quad \text{(S4)}$$

where $\Delta\lambda$ is operating bandwidth, t and μ_i are thickness and initial permeability respectively, and ρ_0 is reflection coefficient.

S5 Split-peak Fit of XRD

The main peaks of the FCC and BCC phases for the samples are too close to each other in the XRD picture, which is not good for calculating the grain size and the phase ratio of the FCC and BCC phases. Firstly, we find the diffraction angles corresponding to the main peaks of FCC and BCC phases with Jade 6 (MDI). Then, we perform split-peak fit by XPS PEAK software.

S6 Supplementary Figures and Tables

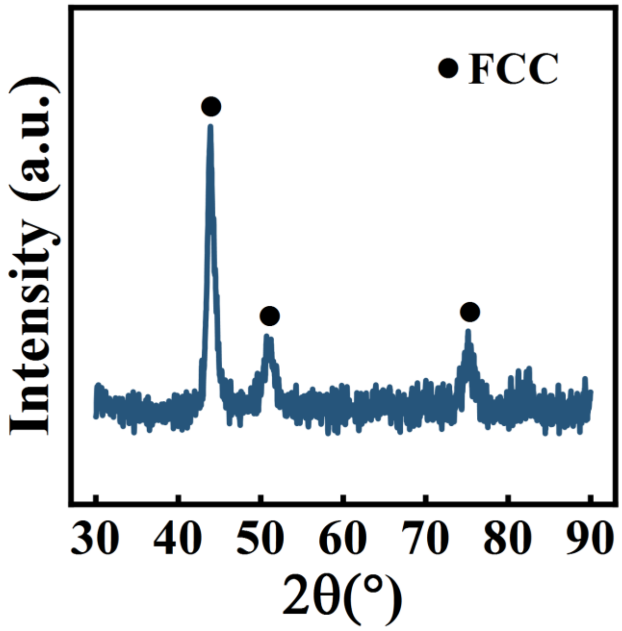

Fig. S1 XRD image of $FeCoNiCr_{0.4}Cu_{0.2}$ HEAs for 20 h dry milling

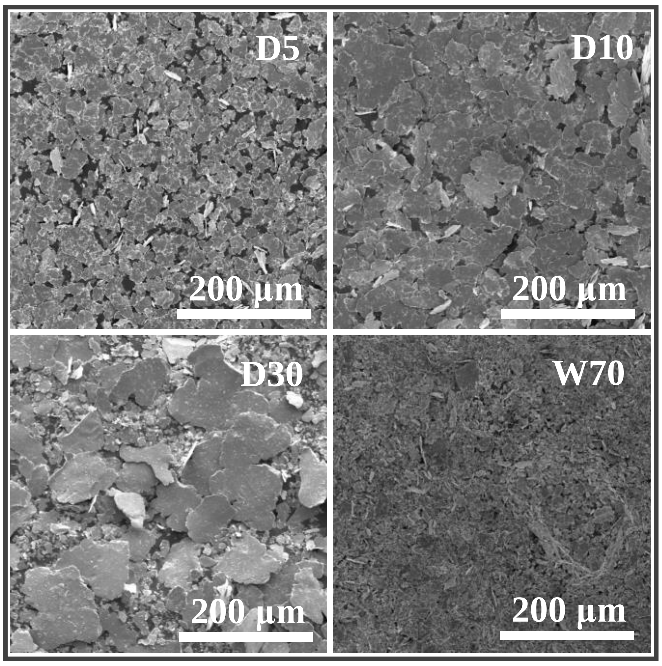

Fig. S2 SEM micrographs of D5, D10, D30, and W70 samples

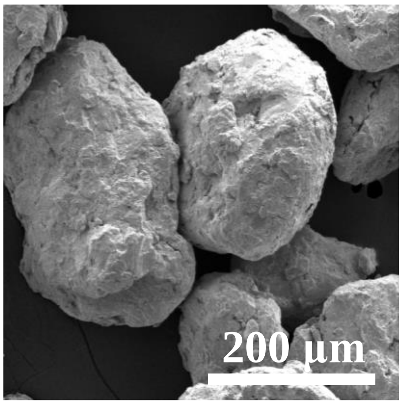

Fig. S3 SEM micrograph of $FeCoNiCr_{0.4}Cu_{0.2}$ HEAs for 20 h dry milling

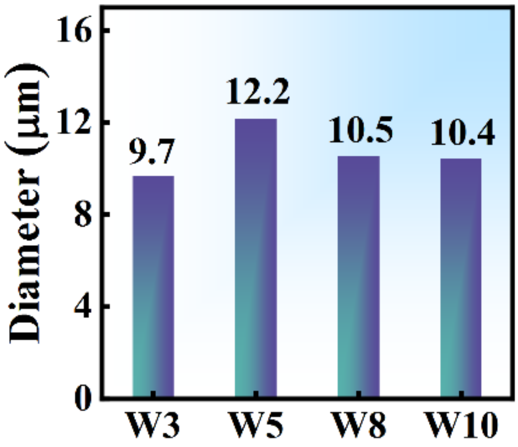

Fig. S4 Length-weighted average diameters of W3, W5 (or D20), W8, and W10 samples

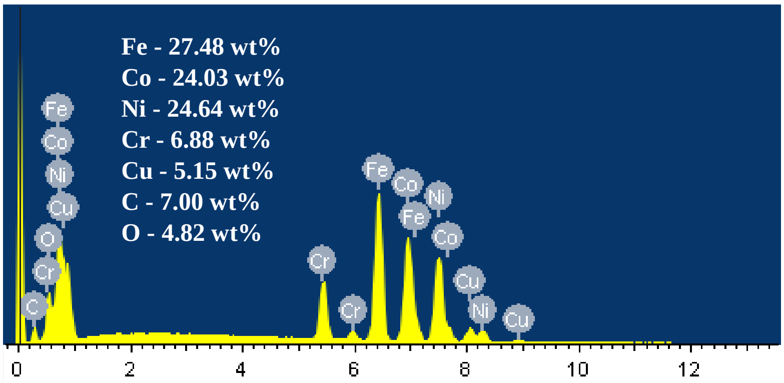

Fig. S5 EDS energy spectrum of W70 sample

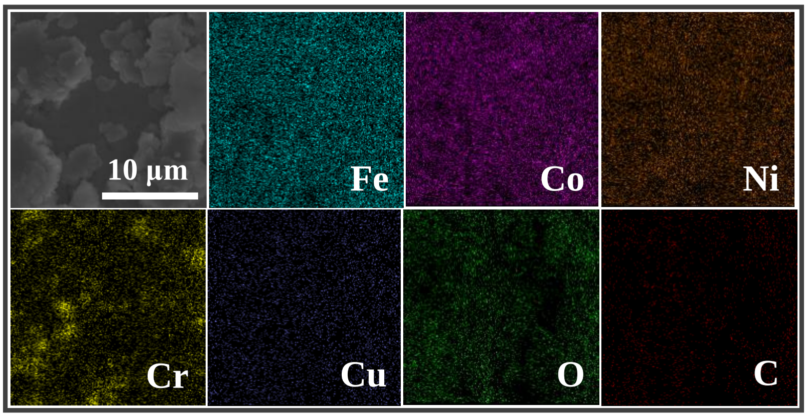

Fig. S6 Elements mapping of W70 sample

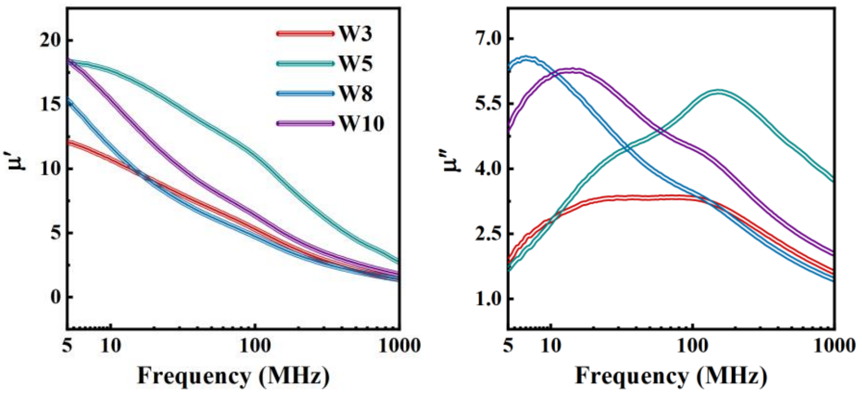

Fig. S7 Real part permeability μ' and imaginary part permeability μ'' of W3, W5 (or D20), W8, and W10 samples in 5−1000 MHz frequency range

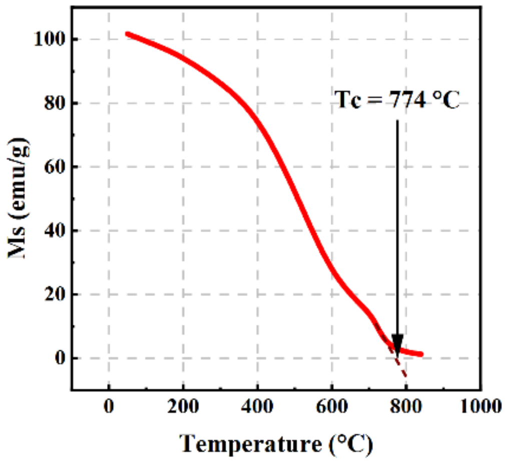

Fig. S8 The thermomagnetic curve of D20 sample from 50 to 840 °C

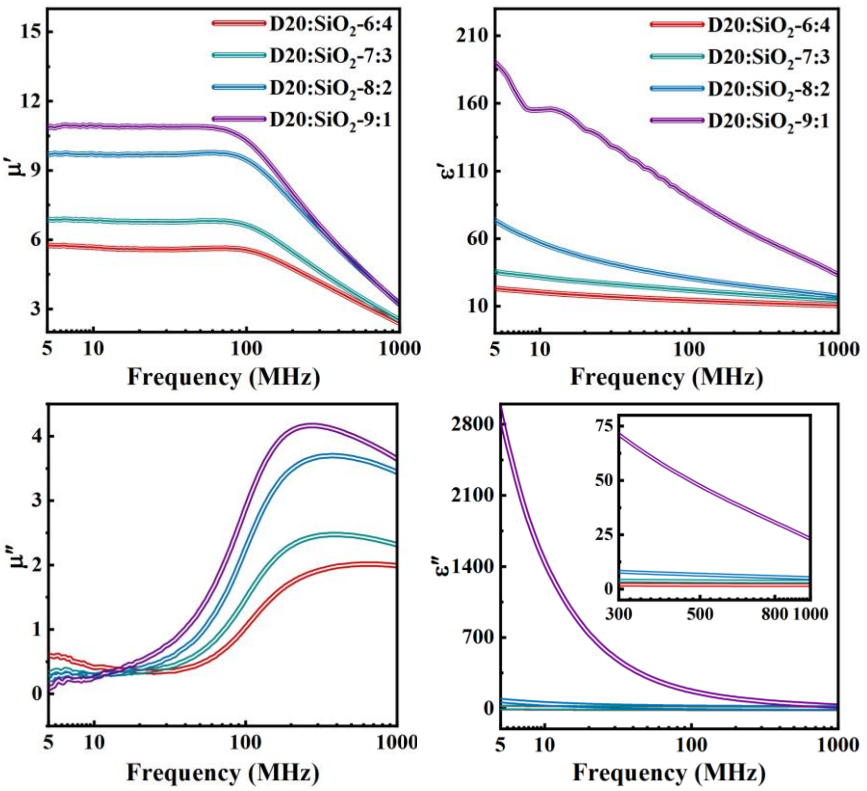

Fig. S9 Complex permeability and complex permittivity of D20/SiO_2 composites with mass ratios of 9:1, 8:2, 7:3, and 6:4 in 5−1000 MHz frequency range at room temperature

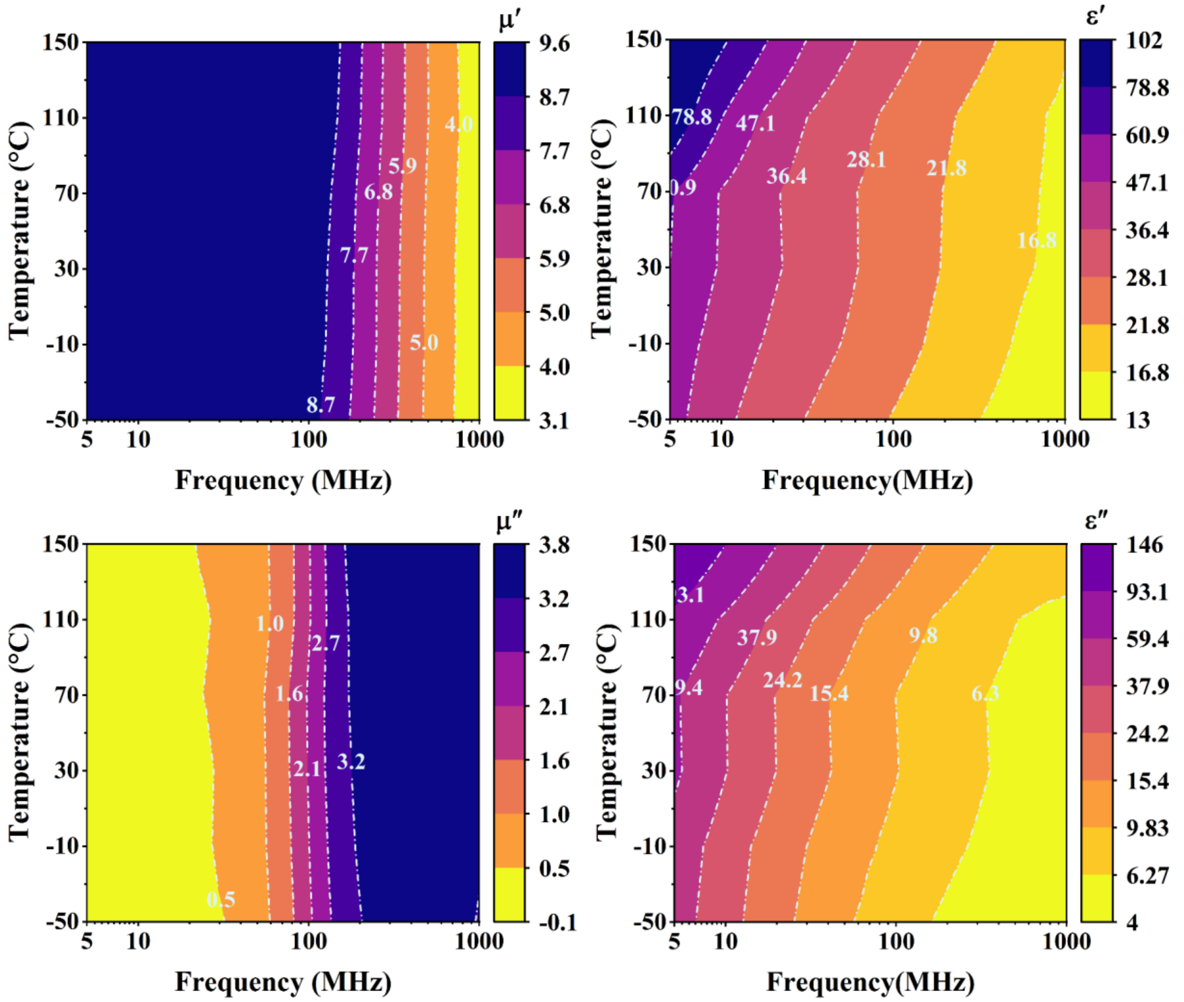

Fig. S10 Complex permeability and complex permittivity of D20/SiO_2 composites with mass ratio of 8:2 in 5−1000 MHz frequency range from −50 to 150 °C

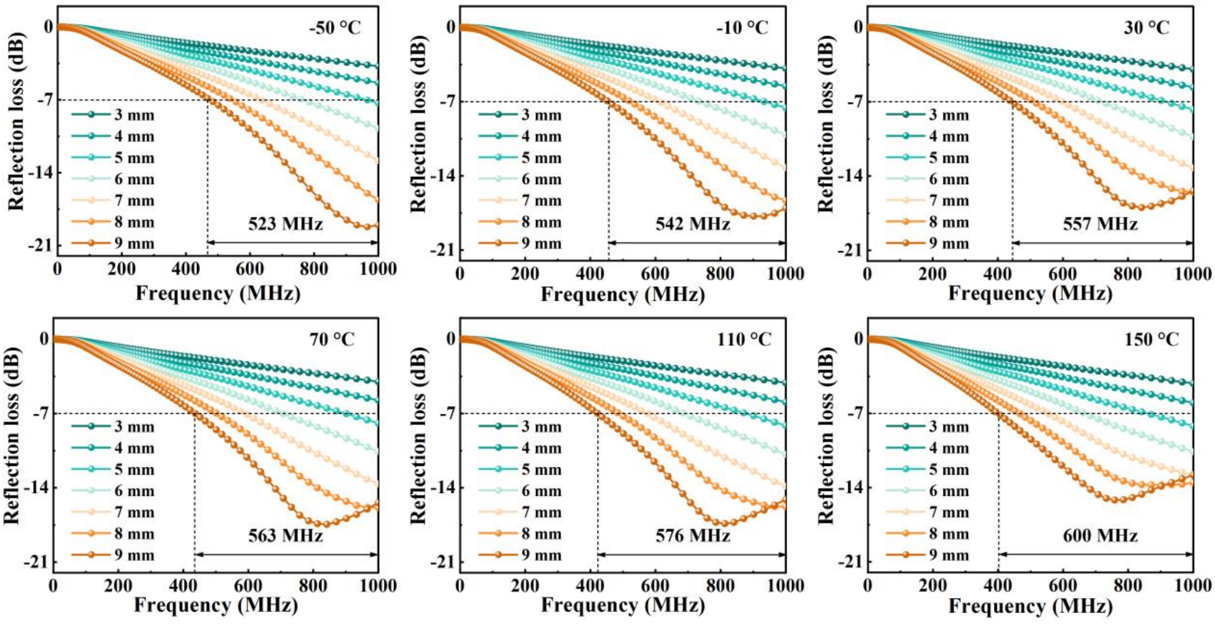

Fig. S11 The reflection loss of D20/SiO_2 composites with mass ratio of 8:2 at different thicknesses from −50 to 150 °C

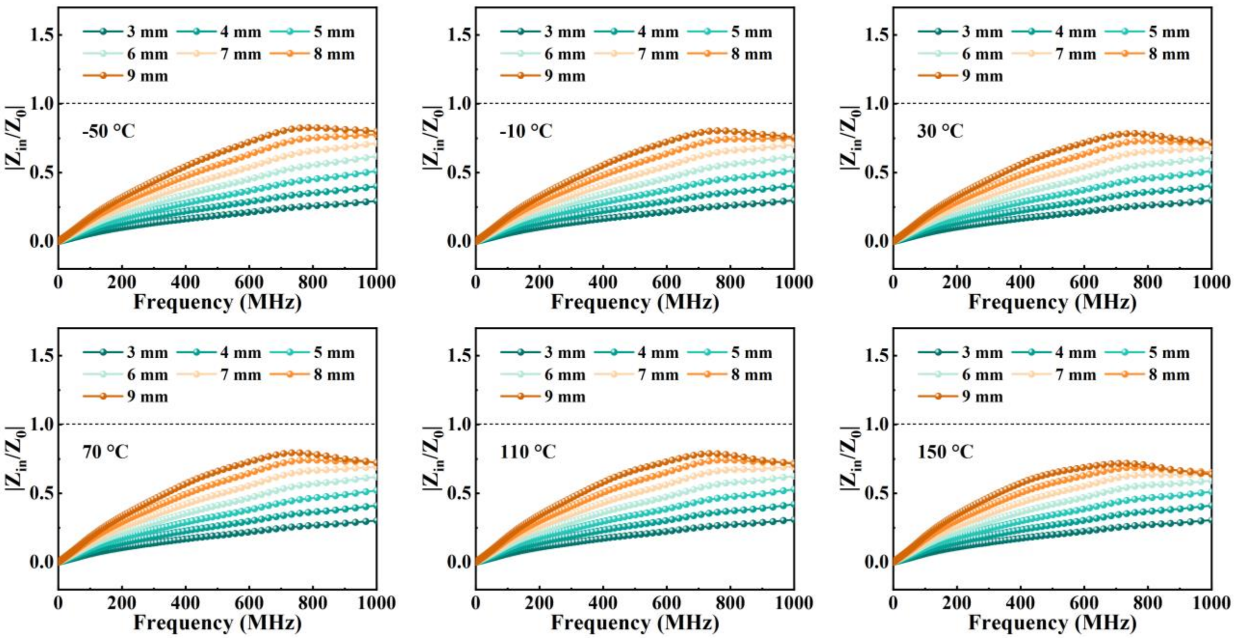

Fig. S12 The impedance matching of D20/SiO_2 composites with mass ratio of 8:2 at different thicknesses from −50 to 150 °C

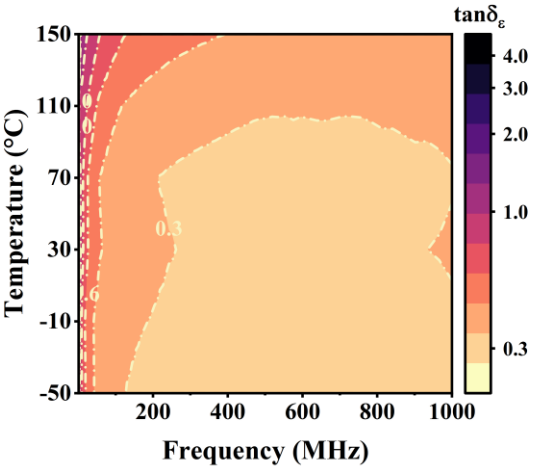

Fig. S13 The dielectric loss tangent of D20/SiO_2 composites with mass ratio of 8:2 from −50 to 150 °C

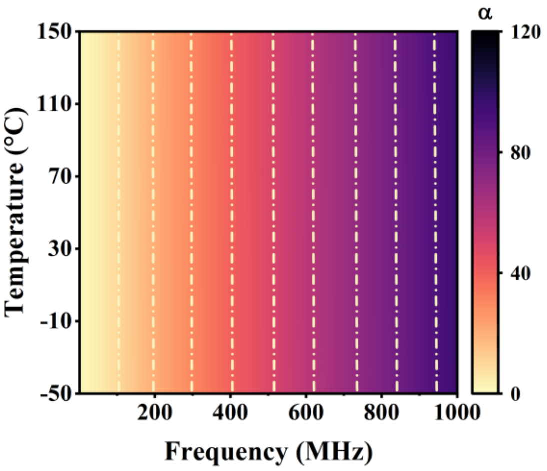

Fig. S14 The attenuation coefficient of D20/SiO_2 composites with mass ratio of 8:2 from −50 to 150 °C

The D20/SiO_2 composites with mass ratio of 8:2 have stable magnetic loss tangent and attenuation coefficient (Fig. S14) with increasing temperature. However, the dielectric loss tangent (Fig. S13) increases sharply with increasing temperature. Therefore, the attenuation coefficient of the D20/SiO_2 composites is mainly determined by the magnetic loss tangent with increasing temperature. The D20/SiO_2 composites with mass ratio of 8:2 have temperature-stable permeability, attenuation coefficient and impedance matching, which results in temperature-stable reflection loss.

Table S1 The particle size, purity, shape and source of the powders about the raw material powders

**Raw materials**	**Fe**	**Co**	**Ni**	**Cr**	**Cu**
Particle size [μm]	50	1–2	< 50	≤ 50	20
Purity	99%	99.5%	99.5%	99.5%	99.8%
Shape	Spherical particles				
Source of the powders	Shanghai Aladdin Bio-Chem Technology Co., LTD				

Table S2 The average complex permeability ($\overline{\mu'}$ and $\overline{\mu''}$) and average complex permeability temperature coefficient ($\overline{\alpha_{\mu'}}$ and $\overline{\alpha_{\mu''}}$) of D5, D10, D20, D30, and W70 samples from −50 to 150 °C at 300 MHz and 1000 MHz, respectively

**Samples**	**300 [MHz]**				**1000 [MHz]**			
	$\overline{\mu'}$	$\overline{\mu''}$	$\overline{\alpha_{\mu'}}$ [°C^{-1}]	$\overline{\alpha_{\mu''}}$ [°C^{-1}]	$\overline{\mu'}$	$\overline{\mu''}$	$\overline{\alpha_{\mu'}}$ [°C^{-1}]	$\overline{\alpha_{\mu''}}$ [°C^{-1}]
D5	2.2	1.6	4.5×10^{-4}	6.5×10^{-4}	1.1	1.1	3.4×10^{-4}	6.4×10^{-4}
D10	5.7	4.8	2.0×10^{-4}	3.3×10^{-4}	2.3	3.3	6.9×10^{-5}	3.4×10^{-4}
D20	6.3	5.2	1.5×10^{-4}	3.0×10^{-4}	2.7	3.8	1.4×10^{-5}	2.6×10^{-4}
D30	2.6	2.5	1.1×10^{-3}	1.1×10^{-3}	1.1	1.4	9.6×10^{-4}	1.4×10^{-3}
W70	5.0	2.5	1.9×10^{-4}	2.8×10^{-4}	2.7	2.7	1.7×10^{-4}	3.0×10^{-4}

Table S3 The comprehensive performance of representative MHz EMW absorbers

**Samples**	**Frequency [MHz]**	**RL_{min} [dB]**	**Absorption bandwidth [MHz]**	**Temperature stability**	**Refs.**
Co_2O_3-doped NiCuZn ferrite-0 wt% Co_2O_3	601	−46	205	/	[S5]
Co_2O_3-doped NiCuZn ferrite-0.5 wt% Co_2O_3	672	−55	234	/	[S5]
Co_2O_3-doped NiCuZn ferrite-1.0 wt% Co_2O_3	778	−51	350	/	[S5]
Co_2O_3-doped NiCuZn ferrite-1.5 wt% Co_2O_3	785	−55	289	/	[S5]
$Ni_{0.5}Co_{0.5}Fe_2O_4$/G	840	−31	420	/	[S6]
Biochar with $FeNi_3$ ferrite	760	−10	240	/	[S7]
NiZn ferrite	617	−21	600	/	[S8]
CIPs/ZnO/G	480	−46	410	/	[S9]
$FeCoNiCr_{0.4}Cu_{0.2}$ HEAs	708	−31	359	Yes	[S10]
(Co/CoO)@C	890	−41	570	/	[S11]
This work (−50 °C)	966	−19	523	Yes	/
This work (−10 °C)	901	−18	542	Yes	/
This work (30 °C)	841	−17	557	Yes	/
This work (70 °C)	841	−17	563	Yes	/
This work (110 °C)	813	−17	576	Yes	/
This work (150 °C)	758	−15	600	Yes	/

Table S4 Comparation of corrosion-resistance performance of different HEA

Samples	Solution	Icorr [μA cm^{-2}]	Ecorr [mV$_{SCE}$]	Refs.
FeCoNiCu	3.5 wt% NaCl	5	−364	[S12]
$Al_2CrFeCoCuTi$	3.5 wt% NaCl	68	−510	[S13]
$Al_2CrFeCoCuTiNi_{0.5}$	3.5 wt% NaCl	32	−430	[S13]
$Al_2CrFeCoCuTiNi_1$	3.5 wt% NaCl	13	−220	[S13]
$Al_2CrFeCoCuTiNi_2$	3.5 wt% NaCl	67	−320	[S13]
$Al_2CrFeCoCuTiNi_{1.5}$	3.5 wt% NaCl	64	−480	[S13]
This work	3.5 wt% NaCl	7	−386	/

Table S5 The fitted electrochemical parameters for the EIS diagram of D20 sample.

Sample	R_s [Ohm]	CPE-T	CPE-P	R_{ct} [Ohm]
D20	133.1	0.00008907	0.6179	3739

Supplementary References

[S1] G. Williamson, W. Hall, X-Ray line broadening fromfiled aluminium and wolfram. Acta Metall. **1**(1), 22-31 (1953). https://doi.org/10.1016/0001-6160(53)90006-6

[S2] X. Liu, Y. Duan, X. Yang, L. Huang, M. Gao et al., Enhancement of magnetic properties in $FeCoNiCr_{0.4}Cu_x$ high entropy alloys through the cocktail effect for megahertz electromagnetic wave absorption. J. Alloy. Compd. **872**, 159602 (2021). https://doi.org/10.1016/j.jallcom.2021.159602

[S3] K.N. Rozanov, Ultimate thickness to bandwidth ratio of radar absorbers. IEEE Trans. Antenn. Propag. **48**, 8 (2000). https://doi.org/10.1109/8.884491

[S4] L. Huang, Y. Duan, X. Dai, Y. Zeng, G. Ma et al., Bioinspired metamaterials: multibands electromagnetic wave adaptability and hydrophobic characteristics. Small **15**(40), 1902730 (2019). https://doi.org/10.1002/smll.201902730

[S5] S. Yan, S. Liu, J. He, H. Luo, L. He et al., Effects of Co_2O_3 on electromagnetic properties of NiCuZn ferrites. J. Magn. Magn. Mater. **452**, 349-353 (2018). https://doi.org/10.1016/j.jmmm.2017.12.108

[S6] P. Yin, Y. Deng, L. Zhang, W. Wu, J. Wang et al., One-step hydrothermal synthesis and enhanced microwave absorption properties of $Ni_{0.5}Co_{0.5}Fe_2O_4$/graphene composites in low frequency band. Ceram. Int. **44**(17), 20896-20905 (2018). https://doi.org/10.1016/j.ceramint.2018.08.096

[S7] P. Yin, L. Zhang, Y. Wang, H. Rao, Y. Wang et al., Combination of pumpkin-derived biochar with nickel ferrite/$FeNi_3$ toward low frequency electromagnetic absorption. J. Mater. Sci. Mater. Electron. **32**, 25698–25710 (2020). https://doi.org/10.1007/s10854-020-04285-8

[S8] L. He, L. Deng, Y. Li, H. Luo, J. He et al., Design of a multilayer composite absorber working in the P-band by NiZn ferrite and cross-shaped metamaterial. Appl. Phys. A **125**(2), 130 (2019). https://doi.org/10.1007/s00339-019-2422-2

[S9] P. Yin, L. Zhang, J. Wang, X. Feng, K. Wang et al., Low frequency microwave absorption property of CIPs/ZnO/Graphene ternary hybrid prepared via facile high-energy ball milling. Powder Technol. **356**, 325-334 (2019). https://doi.org/10.1016/j.powtec.2019.08.033

[S10] X. Liu, Y. Duan, Z. Li, H. Pang, L. Huang et al., $FeCoNiCr_{0.4}Cu_x$ high-entropy alloys with strong intergranular magnetic coupling for stable megahertz electromagnetic absorption in a wide temperature spectrum. ACS Appl. Mater. Interfaces **14**(5), 7012-

7021 (2022). https://doi.org/10.1021/acsami.1c22670

[S11] P. Yin, L. Zhang, Y. Tang, J. Liu, Earthworm-like (Co/CoO)@C composite derived from MOF for solving the problem of low-frequency microwave radiation. J. Alloy. Compd. **881**, 160556 (2021). https://doi.org/10.1016/j.jallcom.2021.160556

[S12] Y.A. Shen, H.M. Hsieh, S.H. Chen, J. Li, S.W. Chen et al., Investigation of FeCoNiCu properties: thermal stability, corrosion behavior, wettability with Sn-3.0Ag-0.5Cu and interlayer formation of multi-element intermetallic compound. Appl. Surf. Sci. **546**, 148931 (2021). https://doi.org/10.1016/j.apsusc.2021.148931

[S13] X. Qiu, C. Liu, Microstructure and properties of $Al_2CrFeCoCuTiNi_x$ high-entropy alloys prepared by laser cladding. J. Alloy. Compd. **553**, 216-220 (2013). https://doi.org/10.1016/j.jallcom.2012.11.100
